# Application of Synchrotron Radiation-Based Fourier-Transform Infrared Microspectroscopy for Thermal Imaging of Polymer Thin Films

**DOI:** 10.3390/polym15030536

**Published:** 2023-01-19

**Authors:** Emigdio Chavez-Angel, Ryan C. Ng, Susanne Sandell, Jianying He, Alejandro Castro-Alvarez, Clivia M. Sotomayor Torres, Martin Kreuzer

**Affiliations:** 1 Catalan Institute of Nanoscience and Nanotechnology (ICN2), CSIC and BIST, The Universitat Autònoma de Barcelona Campus, 08193 Barcelona, Spain; 2 NTNU Nanomechanical Lab, Department of Structural Engineering, Norwegian University of Science and Technology (NTNU), 7491 Trondheim, Norway; 3 Laboratorio de Bioproductos Farmacéuticos y Cosméticos, Centro de Excelencia en Medicina Traslacional, Facultad de Medicina, Universidad de La Frontera, Temuco 4780000, Chile; 4 Institución Catalana de Investigación y Estudios Avanzados (ICREA), 08010 Barcelona, Spain; 5 CELLS-ALBA, Synchrotron Light Source, 08290 Barcelona, Spain

**Keywords:** thermal imaging, synchrotron radiation, machine learning, temperature dependence, FTIR polymer, FTIR thermometry

## Abstract

The thermal imaging of surfaces with microscale spatial resolution over micro-sized areas remains a challenging and time-consuming task. Surface thermal imaging is a very important characterization tool in mechanical engineering, microelectronics, chemical process engineering, optics, microfluidics, and biochemistry processing, among others. Within the realm of electronic circuits, this technique has significant potential for investigating hot spots, power densities, and monitoring heat distributions in complementary metal–oxide–semiconductor (CMOS) platforms. We present a new technique for remote non-invasive, contactless thermal field mapping using synchrotron radiation-based Fourier-transform infrared microspectroscopy. We demonstrate a spatial resolution better than 10 um over areas on the order of 12,000 um^2^ measured in a polymeric thin film on top of CaF_2_ substrates. Thermal images were obtained from infrared spectra of poly(methyl methacrylate) thin films heated with a wire. The temperature dependence of the collected infrared spectra was analyzed via linear regression and machine learning algorithms, namely random forest and k-nearest neighbor algorithms. This approach speeds up signal analysis and allows for the generation of hyperspectral temperature maps. The results here highlight the potential of infrared absorbance to serve as a remote method for the quantitative determination of heat distribution, thermal properties, and the existence of hot spots, with implications in CMOS technologies and other electronic devices.

## 1. Introduction

The temperature and thermal conductivity of a material are among the most common and important physical measurement parameters. Rapid developments in nanotechnology and biological applications have escalated the ever-growing necessity for contactless and non-invasive measurements of these parameters [1,2,3]. Optical measurement techniques such as fluorescence thermometry [4], optical fiber thermometry [5], Raman thermometry [6], and infrared thermometry based on Planck black-body emission [7] are some of the existing techniques that are used to extract the temperature in a remote and non-invasive manner. However, these methods require either very long acquisition times (e.g., Raman thermometry), knowledge of the emissivity which depends on temperature, wavelength and size, or the use of luminescent materials [8].

The extrapolation of temperature from infrared radiation can be achieved by different temperature-dependent features. The most common technique is infrared thermography, in which detecting devices sense the energy radiated from objects in the infrared band region [9]. As the energy emitted by a body is proportional to the fourth power of the temperature rise, the energy values can be converted into the objects surface temperature using Planck’s law of black-body radiation and calibration constants [9]. Infrared thermography is a contactless technique, which can provide two dimensional thermal images in real time [10]. Thermal cameras, based on these principles, can offer spatially resolved thermal images of a sample or scene. The possible applications for such technologies are very broad, finding utility in areas such as the study of irregularities in building envelopes [11], the testing of aerospace components [12], solar cells [13], microelectronic devices [14], or biomedicine [15]. However, determination of the temperature depends on knowledge of the emissivity of the measured surface. The emissivity is a value ranging from zero to one and is dependent on the specific material, surface, and wavelength, and describes the effectiveness of a surface for radiating energy relative to that of a black body [10,15]. In addition to the emissivity, another obstacle to the accurate determination of temperature via infrared thermometry is the complex thermal relationship between a specimen of interest and its surroundings (e.g., convective cooling, ambient humidity, and environmental temperature), which can have a strong effect on measurements [10].

A common approach to obtain thermal maps or local temperatures of a sample is based on Raman spectroscopy. Optothermal Raman or Raman thermometry is one of the most popular and widely used techniques for characterizing the temperature of two-dimensional materials [16], thin films [17,18], substrates [6,19,20], and suspended semiconductors [20,21,22,23,24,25]. Using Raman thermometry, the local temperature can be measured in four different ways: (i) through the ratio of the Stokes and anti-Stokes signal amplitudes and calculation of the temperature based on a Boltzmann distribution of the ground and first excited state populations [26]; (ii) via the analysis of the band position; (iii) linewidth; and (iv) intensity of a Raman mode followed by a determination of the temperature dependence of the associated spectral characteristic [6]. A change in temperature induces changes at the molecular scale, leading to a modification of bond lengths and intermolecular forces. This effect modifies the phonon frequency and its dynamics and gives rise to a shift in the Raman mode frequency (peak position), phonon lifetime (linewidth), and phonon population (amplitude). Consequently, it is possible to extract the local temperature of a sample via the measurement of a Raman band previously calibrated using a reference measurement. Reparaz et al. proposed the measurement of the thermal conductivity via two-laser Raman thermometry. Here, a heating laser creates a temperature gradient in a thin film or membrane and a probing laser locally measures the temperature through Raman imaging [22]. However, Raman thermometry requires the presence of a Raman active mode and can be very time-consuming. Moreover, the use of a laser as a heater may induce the injection of photo-excited charge carriers that can alter the measured properties [27] or induce the overpopulation of nonthermal phonons [28].

While infrared (IR) spectroscopy relies on the same effect of temperature on the phonon modes, IR signals are often stronger than Raman signals and can be calibrated for quantitative measurements using multivariate chemometric methods. Temperature-dependent IR spectra have mainly been employed in the determination of the transition temperature and conformational energy of macromolecules [29,30,31,32]. This method probes subtle changes in the intensities of individual infrared absorbance bands or band ratios with respect to temperature [32,33]. A potential model system for incorporating variable-temperature Fourier-transform infrared (FTIR) spectroscopy is poly(methyl methacrylate) (PMMA), as the temperature dependence of its infrared absorbance band intensities have been studied in detail in recent years [30,34,35]. For example, the conformational energies for the PMMA backbone and side chains were derived by analyzing changes in the infrared absorbance of PMMA as a function of temperature [29,30]. In another work, the glass transition temperature was derived by analyzing changes in the reflectivity of PMMA thin films [31]. Painter et al. studied the thermal transitions of PMMA using the change in width and shape of absorbance bands with increasing temperature [35]. Some PMMA absorbance bands have also been found to be temperature-dependent [29,36].

Due to the stronger signal of IR relative to Raman and the brightness of the synchrotron IR-light source, we propose the exploitation of the temperature dependence of infrared absorbance peaks for thermal imaging in thin films. To our knowledge, the modification of an infrared absorbance band has not been previously used as a metric for the determination of temperature. Thus, we incorporate a simple and fast approach to measure temperature via a linear regression (LR) approach. In addition, we also propose the use of machine learning (ML) algorithms to fully exploit FTIR spectra and apply them to extract temperature information from single peaks or even from a continuous spectral range [37,38]. The development of software, faster computers, and ML enable the application of more general approaches for predicting temperature via measured infrared spectra. In this study, random forests and k-nearest neighbor (k-NN) algorithms were applied to predict the temperature gradient of a test sample using a continuous spectral range. The temperatures predicted by ML were then compared to the LR approach. This work highlights the feasibility of infrared vibrational thermography based on ML approaches and FTIR spectroscopy as a new method for predicting temperatures in polymeric thin films. This approach can be extended to any film with temperature-dependent IR-active modes. Moreover, considering that silicon is transparent to IR excitation, this method could be used to characterize hot spots or power densities in situ.

## 2. Materials and Methods

### 2.1. Sample Preparation

Samples were fabricated on calcium fluoride (CaF_2_) substrates, a common infrared window transparent to IR light. An initial cleaning step was performed by ultrasonication in acetone and isopropanol for 5 min, followed by a deionized water rinse and N_2_ drying. Poly(methyl methacrylate) (PMMA) powder was dissolved in anisole (MicroChem Corp (MA, USA)) using magnetic stirring for more than 12 h in a closed container to avoid solvent evaporation. The powder (with an average molecular weight of 15,000 Da and made by gel permeation chromatography) was purchased from Sigma Aldrich (now Merck KGaA, Darmstadt, Germany). The resulting PMMA solution was spin-coated onto CaF_2_ chips (spin coater from Laurell Technologies) at 4000 rpm and cured at 50 °C for 4 h in a vacuum oven (Binder, Germany). The thickness of the PMMA layer was set to 1 µm. The PMMA layer thicknesses were measured by dual rotating compensator variable angle spectroscopic ellipsometry (VASE) (RC2, J.A. Woollam Co.) over the wavelength range 210–1690 nm with data collected from 55° to 70°.

### 2.2. Metal Wire Preparation and Temperature Calibration

**Figure 1:**
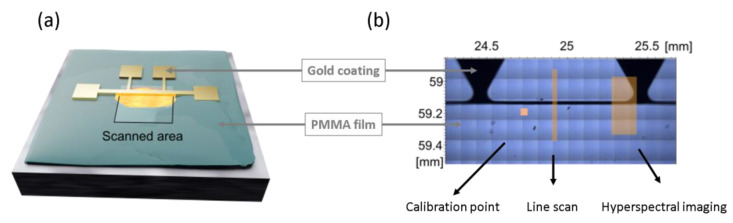
(**a**) Schematic representation of the heating device deposited on top of the polymer film. (**b**) Optical transmission image of the sample, showing the metallic wire in black with the three infrared measurement areas highlighted in orange.

To create a temperature gradient in the PMMA film, a titanium/gold wire was evaporated onto the polymer film and used as a heating element by applying different currents to the wire. The Ti (5 nm) and Au (95 nm) films were deposited using electron-beam evaporation (AJA International, Inc., USA) at a deposition rate of 5 Å/s using a mechanical mask. A schematic illustration is shown in Figure 1a. The deposited metallic strip is composed of four rectangular pads connected by pins to the narrow heating wire. The width of the heating line was set to 20 μm and the length to *l* = 1 mm, the latter being determined by the distance between the inner pads used to measure the voltage. The outer pads were used to inject the current. Since the temperature of the wire is linearly proportional to its electrical resistivity, the temperature can be set by applying the corresponding current/voltage. For the calibration, the electrical resistivity of the wire was measured at different system temperatures ranging between 302 and 350 K on a heating stage to obtain the linear correlation between temperature and wire resistivity (Figure S1). A current source with an integrated voltmeter was connected and the corresponding resistivity was set to use the wire as a heating element.

### 2.3. Synchrotron Radiation-Based Fourier-Transform Infrared (SR-FTIR) Microspectroscopy

Synchrotron radiation-based Fourier-transform infrared (SR-FTIR) microspectroscopy measurements were performed at the infrared microspectroscopy beamline MIRAS of the Spanish synchrotron light source ALBA [39]. The endstation was equipped with a Vertex 70 spectrometer coupled to a Hyperion 3000 visible/infrared microscope (Bruker, Germany). Measurements were carried out in transmission mode using a 15× objective and matching condenser (numerical aperture = 0.4). For each of resulting spectra, 256 scans were co-added for the temperature calibration and 64 scans for the line scans and matrix, with a wavenumber resolution of 4 cm^−1^. The software package Opus (Version 7.5, Bruker, Germany) was used for data acquisition. Data was analyzed, fitted, and plotted using a Python code based on numpy, scipy, and seaborn libraries. Linear baselines were calculated and subtracted in the regions of interest between 2800–3100 cm^−1^ and 950–1800 cm^−1^, respectively. The calibration of PMMA between infrared absorbance and temperature was obtained by using a Linkam FTIR600 temperature control stage mounted in the infrared microscope with two CaF_2_ windows of 500 µm thickness and used in a transmission configuration. The measurements were carried out under atmospheric pressure.

### 2.4. Machine Learning

Machine learning (ML) was performed with the open source software package Orange, Bioinformatics Laboratory of the University of Ljubljana, version 3.30.2, and the spectroscopy add-on, version 0.6.2 [40,41,42]. Within Orange, all spectra were baseline corrected (rubber band) and unit vector normalized in the region of interest after the second derivative was calculated over the total range. In addition, a Gaussian smoothing of the data was applied.

## 3. Results and Discussion

The first step in using polymer IR modes as a thermometer is to calibrate the IR response with respect to temperature. Some variations of the polymer film such as density, molecular weight, or polydispersity index are relevant to the IR response of the film. However, as this method requires an initial calibration of the IR signal against temperature, the impact of changing these properties on the temperature response are already included in the initial calibration. The calibration step is mandatory to be able to exploit an FTIR signal to be used as a thermometer. The infrared absorbance of the sample was locally measured at different regions, as shown in Figure 1b. A single point for calibrating the temperature response of the PMMA IR spectra was repeatedly measured at different environmental temperatures. Here, the sample was placed in a cryostat stage and no current was applied to the metal wire. Subsequently, the sample was heated using the metal wire and the cryostat stage was kept at room temperature. In this configuration, the infrared response of the PMMA layer was measured along a line scan across the heated metal wire and across a larger area for hyperspectral imaging.

### 3.1. PMMA Infrared Absorption (Calibration Data)

**Figure 2:**
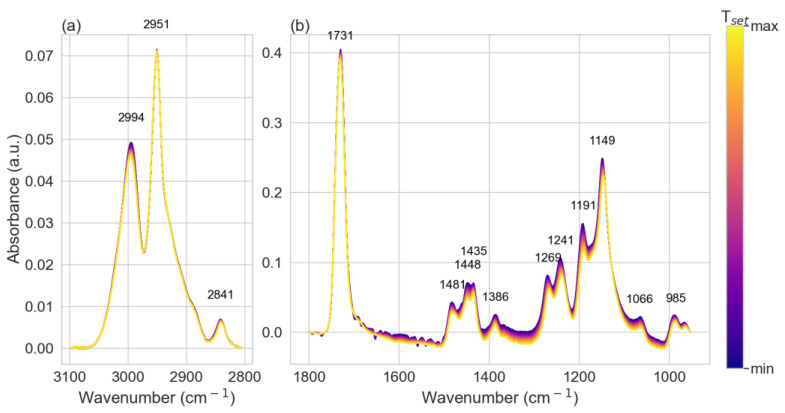
Infrared absorption spectra of PMMA measured with variable temperature from 283 and 373 K in 3 K steps. (**a**) All spectra have been baseline corrected in the region of the C-H vibrational peaks between 3100 and 2800 cm^−1^ and (**b**) the fingerprint region between 1800 and 950 cm^−1^.

For the calibration of the infrared absorbance response of PMMA at different temperatures, spectra of the PMMA film were systematically taken at several set temperatures (T_set_) using a cryostat stage (Linkam FTIR600). Measurements were taken between 283 and 373 K in 3 K steps during the heating and subsequent cooling cycle. A total of 5 min passed following each change in temperature prior to spectrum acquisition to allow for thermal equilibration. Infrared absorbance spectra of PMMA are shown in the C-H stretch region between 3100–2800 cm^−1^ (Figure 2a) and the fingerprint region between 1800 and 950 cm^−1^ (Figure 2b). All absorption peaks were identified and associated to the molecular vibrational modes according to the literature and were compared with density-functional theory (DFT) simulations (Figure S2) [43,44,45,46,47,48]. The room temperature band assignments are summarized in Table 1.

As T_set_ was increased, the absorbance of the sample decreased systematically for all wavenumbers. The decrease in amplitude with increasing temperature was caused by a combination of different temperature-dependent phenomena such as intermolecular interactions, changes in conformational populations, Fermi resonance, and the dynamics of molecular groups [30]. A systematic change in the Gaussian amplitude, linewidth (full width half maximum, FWHM), and band position is observed in Figures S3–S5 (from the fitting), in the two-dimensional correlation map (Figure S6), and in simulation (Figure S7) in the Supplementary Materials [54,55].

**Figure 3:**
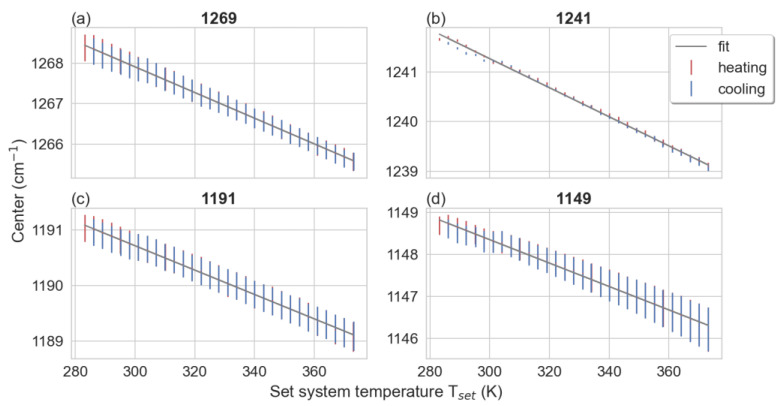
Temperature dependence of different bands located at: (**a**) 1269, (**b**) 1241, (**c**) 1191, and (**d**) 1149 cm^−1^. The sample has been heated (red) and cooled (blue) over the temperature region between 283 and 373 K. Data points are shown with numerical error from the fit. A linear model has been fitted (grey lines) with the resulting slopes listed in Table 1.

A temperature-dependent shift in peak positions was also observed. The PMMA absorbance peaks listed in Table 1 were individually fitted with Gaussian line shapes (Figure S8). To more accurately determine the peak position, the fits were performed only around the peak maxima, typically ± 10 cm^−1^. The resulting fitting parameters were the maximum peak position (peak), amplitude, and FWHM. The temperature dependence of all three fitting parameters were plotted against temperature (Figure S3–S5). The impact of the change of all these parameters in the band around 1149 cm^−1^ is shown in Figure S6 using two-dimensional correlation spectroscopy (2DCOS). The synchronous plot (Figure S6a) shows the typical asymmetrical four-leafed clover pattern which indicates that the redshift of the peak is strongly coupled with peak broadening and decreasing intensity as temperature increases. The effect of each parameter and its coupling with the measured signal is also shown in the simulated 2DCOS in Figure S7. A steeper slope corresponds to a more pronounced temperature dependence for the given absorbance band. The IR bands exhibiting the strongest temperature variations are those at 1269, 1241, 1191, and 1149 cm^−1^, with temperature dependencies of the peak center positions of 0.032, −0.029, −0.022, and −0.028 cm^−1^/K, respectively (Figure 3). These peaks arising from the monomer backbone units can then be used for the prediction of temperature on a test sample.

The temperature dependencies of the most temperature sensitive peaks (1269, 1241, 1191, and 1149 cm^−1^) are shown in Figure 3. All four peaks possess a linear relationship between T_set_ and the infrared peak position (IPP) over the measured temperature range. The resulting temperature dependencies (the slope of the linear fit *dIPP/dTset*) for all peaks are shown in Figures S8 and S9; they have been summarized in Table 1, and are used for the LR approach for the temperature determination. The pronounced redshift of the peaks at 1269, 1241, 1191, and 1149 cm^−1^ have been previously reported and are in good agreement with our results (Figure 3) [29,36].

### 3.2. Line Scans (Test Sample)

Following IR calibration, a proof-of-concept experiment was performed by heating the same film but by using a gold heater wire. The wire was heated by a DC current through the Joule effect and then thermal mapping was realized by measuring the infrared absorbance spectra at different positions along the polymer. The first measurements were taken along line scans at positions from −200 to 200 µm relative to the center of the gold wire (0.5 mm away from the voltage pad) at 5 µm steps with a sampled area of 10 × 10 µm^2^ for each spectrum. All the mapping lines were taken inside of the cryostat with an environmental temperature set to 318 K. For each line scan, the wire temperature (T_wire_) of the wire was left at room temperature (298 K, baseline) or heated to 334 K, 353 K, or 376 K. We then calculated the temperature distribution of the PMMA at each measurement position using linear regression and machine learning.

#### 3.2.1. Temperature Prediction by Linear Regression Model

**Figure 4:**
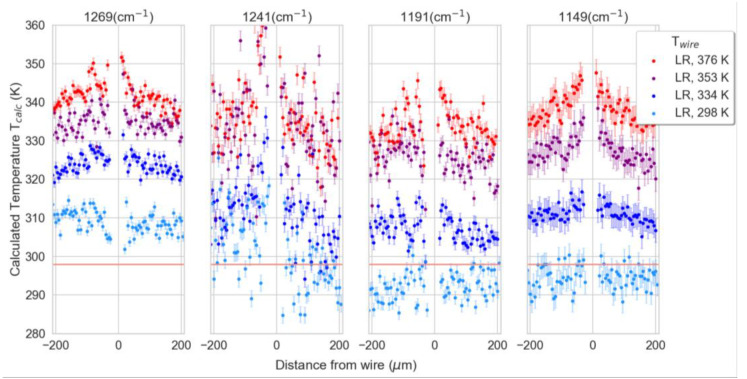
Calculated sample temperatures (T_calc_) at +/− 200 µm from the heating wire (x = 0), using the peaks at 1269, 1241, 1191, and 1149 cm^−1^, including error bars. For each measurement, the wire temperature (T_wire_) was left at room temperature (298 K, light blue) or heated to 334 K (blue), 353 K (purple), or 376 K (red). Prior to each measurement, the wire temperature was given at least 10 min to equilibrate. The room temperature inside of the cryostat is indicated with horizontal red lines.

Figure 4 shows the four selected peaks with the highest temperature responses (1269, 1241, 1191, and 1149 cm^−1^) which were selected and used to calculate the temperature at each measurement position by using the linear temperature dependence of the absorbance peak positions measured in the calibration step (Figure 3). Here, the peak positions alone were used as predictors of the temperature, carried out separately for each peak.

First, the calculated temperatures (T_calc_) for the measurements at room temperature (298 K) were compared to the set wire temperature (T_wire_). The mean temperatures for each of these room temperature measurement points were calculated. The resulting mean values (given with standard derivation) are 309 ± 3, 302 ± 8, 291 ± 4, and 294 ± 3 K for the peaks at 1269, 1241, 1191, and 1149 cm^−1^, respectively.

As expected, T_calc_ calculated for higher wire temperatures (T_wire_) increased with increasing T_wire_. A temperature gradient can be observed with the maximum temperatures close to the wire positions which decays exponentially away from these positions. The decay of the thermal field is given by [56]:

(1) $$\Delta T=Q\sqrt{\frac{1}{hkd}}\exp\left[-\sqrt{\frac{h}{kd}}x\right]$$

where *Q* is power density of the wire, *h* is the “convection coefficient” (which is the thermal boundary conductance (TBC) between the polymer and the substrate in this case), *d* is the film thickness, and *k* is the thermal conductivity of the polymer. From the best fit of (1) (Figure S10), the thermal conductivity and interphase thermal resistance were estimated to be *k* = 0.28 ± 0.11 WK^−1^m^−1^ and TBC *h* = 85.30 ± 0.15 × 10^6^ WK^−1^m^−2^, showing good agreement with the expected values for the thermal conductivity 0.15 < *k* < 0.25 (WK^−1^m^−1^) [57], and the same order for the TBC 60 < *h* < 150 × 10^6^ WK^−1^m^−2^ for the case of PMMA-metal [58]. An extended description of the fitting procedure is provided in the Supplementary Materials.

#### 3.2.2. Temperature Prediction by Machine Learning Approach

In addition to the simple linear regression approach, the measured calibration data enabled the data to be analyzed with ML approaches. Despite the simplicity of linear regression, an important advantage of ML is that multiple complex spectral regions can be simultaneously considered to determine the temperature, rather than only the position of a single peak, as carried out previously in the linear regression approach. Another advantage is that no further knowledge about the temperature sensitivity of individual peaks is necessary and, therefore, the calibration data does not have to be analyzed beforehand. Moreover, the full spectra can contain subtle temperature-dependent variations that are invisible to the naked eye but detectable by ML. This approach begins with an unsupervised principal component analysis (PCA), applied to identify the temperature-sensitive spectral regions of interest in the fingerprint region from 1550 to 1000 cm^−1^ (e.g., Figure 2b) for processed second derivative spectra. The resulting scores plot (Figure S11a) shows a clear separation between the line scan data taken at different temperatures (circles) along principal component 2 (PC-2). In addition, the calibration data (stars) also separate temperature dependence along PC-2 with measurements at higher temperatures resulting in a positive PC-2 and those at lower temperatures resulting in a negative PC-2. Consistent with this result, the same trend along PC-2 has been observed for the line scan data.

**Figure 5:**
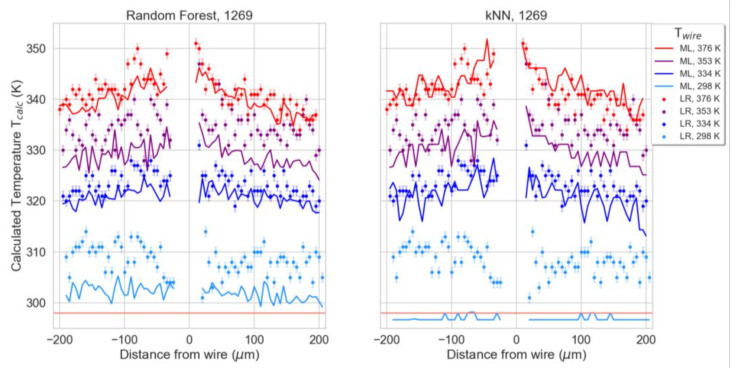
Temperatures (T_calc_) predicted with linear regression (LR) for the peak at 1269 cm^−1^ indicated by “●”-markers and temperatures predicted by the machine learning (ML) approaches indicated by lines, using the random forest (**left**) and k-NN (**right**) algorithms. The data were measured with the gold wire temperature (Twire) at room temperature (298 K) and heated to 334, 353, and 376 K. Room temperature is indicated with horizontal red lines.

The wavenumber regions with higher intensities of PC-2 (Figure S11b) were used for the following supervised analysis. We identified two spectral regions of interest from 1510–1410 cm^−1^ and 1300–1120 cm^−1^, and we discarded the other wavenumber regions. The pre-processed second derivative spectra were baseline corrected and unit vector normalized before further analysis. The random forest and k-nearest neighbor (k-NN) algorithms were applied and compared as supervised models, using the calibration spectra as training data to predict the temperature of the test sample spectra taken close to the heated wire. A total of 1000 trees and 20 neighbors were chosen for the random forest and k-NN algorithms with a Euclidian metric, respectively. The cross validation of the models using 20 folds resulted in high coefficients of determination (R2) of 0.999 and 0.952 and low mean absolute errors (MAE) of 0.691 and 3.477 for the random forest and k-NN algorithms, respectively. The temperatures predicted by the machine learning approaches are plotted in Figure 5 for the two models separately for each applied wire temperature. The temperatures calculated via linear regression for the peak at 1269 cm^−1^ from Figure 4 are also overlaid onto these plots for direct comparison.

### 3.3. Hyperspectral Imaging

**Figure 6:**
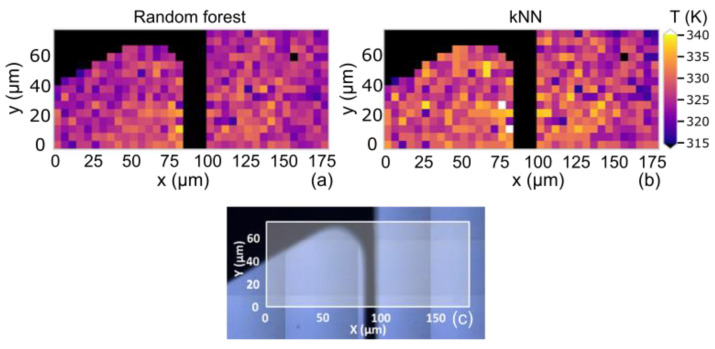
Hyperspectral images showing the temperatures predicted by the (**a**) random forest and (**b**) k-NN machine learning algorithms. The regions of the metallic gold wire are masked in black. (**c**) Optical image with the metallic gold coating (black) on a PMMA thin film (blue) and the sampled area of the hyperspectral imaging in white.

After measuring the line scan in the center of the heater line, we moved to a corner of a voltage pad to take a snapshot of the temperature profile. Measurements at this location were taken due to the possibility of generating non-homogeneous maps due to the influence of the voltage pad. Here, a matrix of 36 × 15 spectra (540) was measured with a step size of 5 µm and a sampled area of 10 × 10 um^2^ per spectrum (Figure 6c). The black region in the image is due to a mask over the heating wire, which blocked the transmitted IR light.

Here, we set the wire temperature to 376 K, measured by the resistance of the wire. For each measurement point, the temperature was predicted using the random forest (Figure 6a) and the k-NN (Figure 6b) algorithms. As previously observed in the line scan predictions (Figure 5), the temperatures predicted by the random forest algorithm were slightly lower than those predicted by the k-NN algorithm. However, this technique allows for a very quick and direct generation of a thermal map using the raw signal, avoiding the individual fitting of all 540 spectra while considering the entirety of the frequency windows rather than a single peak.

## 4. Summary

In this work, we presented a novel contactless technique for thermal mapping based on FTIR spectroscopy. For this approach, the temperature dependence of an IR signal of a 1 µm PMMA thin film was used as a local thermometer with a spatial resolution limited by the spot size of the IR source (10 µm in this work). We incorporated machine learning methods to directly treat measured raw signals from a sample, avoiding the need for peak fitting and analysis of each individual spectrum. An important advantage of ML is that multiple complex spectral regions can be simultaneously considered to determine the temperature, rather than only the position of a single peak. The full spectra can contain subtle temperature-dependent variations that are invisible to the naked eye but detectable by ML. Another advantage is that no further knowledge about the temperature sensitivity of individual peaks is necessary and, therefore, calibration data does not need to be analyzed beforehand. This approach can be extended and adapted to any spectroscopic technique (e.g., Raman, photoluminescence, Brillouin, etc.) and any film can be used as a surface thermometer, as long as it exhibits a temperature-dependent spectroscopic signal. The use of FTIR spectroscopy itself allows for investigation of temperature distributions in CMOS technology, as silicon is transparent and invisible in the IR regime. This opens the possibility to detect hot spots in normal device-operating conditions if they are covered with an IR-active material. This new approach is a step towards a deeper understanding of in situ thermal management in electronic devices that can be performed under operating conditions.

## Figures and Tables

**Table 1:** Peak assignment of the measured PMMA absorption peaks compared to literature values at room temperature.

Vibrational Mode	Fitted Peaks [cm^−1^]	Lit. Values [cm^−1^]	Ref.	Slope (dIPP/dT_set_) [cm^-1^/K]
C-H stretch region:				
C–H stretching vibrations of the –CH_3_	2995	2997	[49]	0.007
C–H stretching vibrations of the –CH_2_	2951	2952	[49]	0.001
methoxy carbon (O–CH3)	2841	2841.6	[50]	−0.013
Fingerprint region:				
C=O stretch (Ketone)	1731	1727, 1732	[49,50,51]	0.001
CH_2_ bending	1481	1485	[52]	−0.014
C–H bending (Alkane)	1448	1450, 1444	[49,51]	0.000
asymmetrical bending vibration (CH_3_)	1434	1435.3	[50]	−0.008
methyl group (C-CH_3_)	1386	1388	[49]	0.009
C-C-O bending	1269	1265	[52,53]	−0.032
C-C-O bending	1241	1238	[52,53]	−0.029
CH3 wagging	1191	1197	[52]	−0.022
C-O-C	1149	1149	[49,50,51]	−0.028
stretching vibration of C–O–C group	1066	1063.6	[49,50]	−0.007
C-C stretching	985	990	[52]	−0.017

## Data Availability

Raw data can be provided by the corresponding author (M.K.) on reasonable request.

## Supplementary Materials

The following supporting information can be downloaded at: https://www.mdpi.com/article/10.3390/polym15030536/s1, Figure S1: electrical resistivity of the gold wire measured at different temperatures in a heating stage. Figure S2: simulated IR spectra of PMMA molecules. Figure S3: fitted Gaussian center positions for each PMMA absorbance peak vs temperature. Figure S4: fitted Gaussian amplitudes for each PMMA absorbance peak vs temperature. Figure S5: fitted Gaussian full width at half maximum (FWHM) for each PMMA absorbance peak vs temperature. Figure S6: synchronous (a) and asynchronous (b) two-dimensional correlation spectra obtained from temperature-dependent FTIR spectra of the PMMA film. The red and blue colors represent positive and negative cross peaks, respectively. Figure S7: synchronous two-dimensional correlation spectra obtained from simulated spectra centered at ~1149 cm^−1^. (a) Simulated 2DCOS fixing band position and linewidth and varying the intensity. (b) Simulated 2DCOS fixing band intensity and linewidth and varying the peak position. (c) Simulated 2DCOS fixing band position and intensity and varying the linewidth. (d) Simulated 2DCOS varying band position, linewidth, and intensity. Figure S8: PMMA absorbance peaks at 283 K fitted individually using a Gaussian line shape, showing the data points and the fit as black lines. Figure S9: fitting results for a linear fit to the temperature dependence of the peak center positions for different absorbance peaks. The linear fits are shown in red in Figure S1. Figure S10: thermal decay as function of sample position for different temperatures of the heater wire. The solid lines represent the best fit. Figure S11: (a) Transferred data (scores plot) of the PCA with the calibration data (stars) and the line scan data points (circle). (b) Loadings plot of the PCA showing PC-1 (black) and PC-2 (red). Figure S12: simplified workflow of the machine learning approach in this work performed by Orange.

## References

[1] Kim M.M., Giry A., Mastiani M., Rodrigues G.O., Reis A., Mandin P. (2015). Microscale thermometry: A review. Microelectron. Eng..

[2] McCabe K.M., Hernandez M. (2010). Molecular thermometry. Pediatr. Res..

[3] Mehboudi M., Sanpera A., Correa L.A. (2019). Thermometry in the quantum regime: Recent theoretical progress. J. Phys. A Math. Theor..

[4] Chihara T., Umezawa M., Miyata K., Sekiyama S., Hosokawa N., Okubo K., Kamimura M., Soga K. (2019). Biological Deep Temperature Imaging with Fluorescence Lifetime of Rare-Earth-Doped Ceramics Particles in the Second NIR Biological Window. Sci. Rep..

[5] Katsumata T., Morita K., Komuro S., Aizawa H. (2014). Fiber-optic thermometer application of thermal radiation from rare-earth end-doped SiO2 fiber. Rev. Sci. Instrum..

[6] Jaramillo-Fernandez J., Chavez-Angel E., Sotomayor-Torres C.M. (2018). Raman thermometry analysis: Modelling assumptions revisited. Appl. Therm. Eng..

[7] Lahiri B.B., Bagavathiappan S., Jayakumar T., Philip J. (2012). Medical applications of infrared thermography: A review. Infrared Phys. Technol..

[8] Childs P.R.N., Dias Carlos L., Palacio F. (2015). Chapter 1. Nanoscale Thermometry and Temperature Measurement. Thermometry at the Nanoscale: Techniques and Selected Applications.

[9] Meola C., Carlomagno G.M. (2004). Recent advances in the use of infrared thermography. Meas. Sci. Technol..

[10] Usamentiaga R., Venegas P., Guerediaga J., Vega L., Molleda J., Bulnes F.G. (2014). Infrared thermography for temperature measurement and non-destructive testing. Sensors.

[11] Kirimtat A., Krejcar O. (2018). A review of infrared thermography for the investigation of building envelopes: Advances and prospects. Energy Build..

[12] Meola C., Boccardi S., Carlomagno G.M., Jawaid M., Thariq M. (2018). Composite material overview and its testing for aerospace components. Sustainable Composites for Aerospace Applications.

[13] Herraiz Á.H., Marugán A.P., Márquez F.P.G. (2020). A review on condition monitoring system for solar plants based on thermography. Non-Destructive Testing and Condition Monitoring Techniques for Renewable Energy Industrial Assets.

[14] May D., Wunderle B., Ras M.A., Faust W., Gollhard A., Schacht R., Michel B. Material characterization and non-destructive failure analysis by transient pulse generation and IR-thermography. Proceedings of the 14th International Workshop on THERMal INvestigation of ICs and Systems, THERMINIC 2008.

[15] Szentkuti A., Kavanagh H.S., Grazio S. (2011). Infrared thermography and image analysis for biomedical use. Period. Biol..

[16] Malekpour H., Balandin A.A. (2018). Raman-based technique for measuring thermal conductivity of graphene and related materials. J. Raman Spectrosc..

[17] Huang S., Chen Y., Luo Z., Xu X. (2021). Temperature and Strain Effects in Micro-Raman Thermometry for Measuring In-Plane Thermal Conductivity of Thin Films. Nanoscale Microscale Thermophys. Eng..

[18] Xu D., Sang Y., Chu Y., Yu Y., Liu F., Hou Y., Wang X. (2021). Optothermal Raman measurement determined thermal conductivity characteristics in NiMn2O4 films grown by chemical solution deposition. Mater. Res. Express.

[19] Wang H., Thomas J., Okuniewski M.A., Tomar V. (2020). Microstructure dependent thermal conductivity measurement of Zircaloy-4 using an extended Raman thermometry method. J. Nucl. Mater..

[20] Stoib B., Filser S., Stötzel J., Greppmair A., Petermann N., Wiggers H., Schierning G., Stutzmann M., Brandt M.S. (2014). Spatially resolved determination of thermal conductivity by Raman spectroscopy. Semicond. Sci. Technol..

[21] Yang L., Prasher R., Li D. (2021). From nanowires to super heat conductors. J. Appl. Phys..

[22] Reparaz J.S., Chavez-Angel E., Wagner M.R., Graczykowski B., Gomis-Bresco J., Alzina F., Sotomayor Torres C.M. (2014). A novel contactless technique for thermal field mapping and thermal conductivity determination: Two-laser Raman thermometry. Rev. Sci. Instrum..

[23] Sett S., Aggarwal V.K., Singha A., Raychaudhuri A.K. (2020). Temperature-dependent Thermal Conductivity of a Single Germanium Nanowire Measured by Optothermal Raman Spectroscopy. Phys. Rev. Appl..

[24] Soini M., Zardo I., Uccelli E., Funk S., Koblmüller G., Fontcuberta i Morral A., Abstreiter G. (2010). Thermal conductivity of GaAs nanowires studied by micro-Raman spectroscopy combined with laser heating. Appl. Phys. Lett..

[25] Cong X., Liu X.L., Lin M.L., Tan P.H. (2020). Application of Raman spectroscopy to probe fundamental properties of two-dimensional materials. npj 2D Mater. Appl..

[26] McGrane S.D., Moore D.S., Goodwin P.M., Dattelbaum D.M. (2014). Quantitative tradeoffs between spatial, temporal, and thermometric resolution of nonresonant Raman thermometry for dynamic experiments. Appl. Spectrosc..

[27] Liao B., Maznev A.A., Nelson K.A., Chen G. (2016). Photo-excited charge carriers suppress sub-terahertz phonon mode in silicon at room temperature. Nat. Commun..

[28] Gallego Lluesma E., Mendes G., Arguello C.A., Leite R.C.C. (1974). Very high non-thermal equilibrium population of optical phonons in GaAs. Solid State Commun..

[29] Tretinnikov O.N., Ohta K. (2002). Conformation-sensitive infrared bands and conformational characteristics of stereoregular poly(methyl methacrylate)s by variable-temperature FTIR spectroscopy. Macromolecules.

[30] O’Reilly J.M., Mosher R.A. (1981). Conformational Energies of Stereoregular Poly(methyl methacrylate) by Fourier Transform Infrared Spectroscopy. Macromolecules.

[31] Shin H.S., Jung Y.M., Oh T.Y., Chang T., Bin Kim S., Lee D.H., Noda I. (2002). Glass transition temperature and conformational changes of poly(methyl methacrylate) thin films determined by a two-dimensional map representation of temperature-dependent reflection-absorption FTIR spectra. Langmuir.

[32] Mahendia S., Heena, Kandhol G., Deshpande U.P., Kumar S. (2016). Determination of glass transition temperature of reduced graphene oxide-poly(vinyl alcohol) composites using temperature dependent Fourier transform infrared spectroscopy. J. Mol. Struct..

[33] Zhang Y., Zhang J., Lu Y., Duan Y., Yan S., Shen D. (2004). Glass Transition Temperature Determination of Poly(ethylene terephthalate) Thin Films Using Reflection-Absorption FTIR. Macromolecules.

[34] Havriliak S., Roman N. (1966). The infra-red absorption characteristics of syndiotactic poly(methyl methacrylate) from 1050 cm-1 to 1300 cm^−1^. Polymer.

[35] Painter P., Zhao H., Park Y. (2011). Infrared spectroscopic study of thermal transitions in poly(methyl methacrylate). Vib. Spectrosc..

[36] Dybal J., Štokr J., Schneider B. (1983). Vibrational spectra and structure of stereoregular poly(methyl methacrylates) and of the stereocomplex. Polymer.

[37] Gomes Rios T., Larios G., Marangoni B., Oliveira S.L., Cena C., Alberto do Nascimento Ramos C. (2021). FTIR spectroscopy with machine learning: A new approach to animal DNA polymorphism screening. Spectrochim. Acta Part A Mol. Biomol. Spectrosc..

[38] Enders A.A., North N.M., Fensore C.M., Velez-Alvarez J., Allen H.C. (2021). Functional Group Identification for FTIR Spectra Using Image-Based Machine Learning Models. Anal. Chem..

[39] Yousef I., Ribó L., Crisol A., Šics I., Ellis G., Ducic T., Kreuzer M., Benseny-Cases N., Quispe M., Dumas P. (2017). MIRAS: The Infrared Synchrotron Radiation Beamline at ALBA. Synchrotron Radiat. News.

[40] Toplak M., Read S.T., Sandt C., Borondics F., Vaccari L., Byrne H.J., Wrobel T.P. (2021). Quasar: Easy Machine Learning for Biospectroscopy. Cells.

[41] Demsar J., Curk T., Erjavec A., Gorup C., Hocevar T., Milutinovic M., Mozina M., Polajnar M., Toplak M., Staric A. (2013). Orange: Data Mining Toolbox in Python. J. Mach. Learn. Res..

[42] Toplak M., Birarda G., Read S., Sandt C., Rosendahl S.M., Vaccari L., Demšar J., Borondics F. (2017). Infrared Orange: Connecting Hyperspectral Data with Machine Learning. Synchrotron Radiat. News.

[43] Forte M., Silva R., Tavares C., Silva R. (2021). Is Poly(methyl methacrylate) (PMMA) a Suitable Substrate for ALD?: A Review. Polymers.

[44] Zhao Y., Schultz N.E., Truhlar D.G. (2006). Design of Density Functionals by Combining the Method of Constraint Satisfaction with Parametrization for Thermochemistry, Thermochemical Kinetics, and Noncovalent Interactions. J. Chem. Theory Comput..

[45] Ditchfield R., Hehre W.J., Pople J.A. (1971). Self-Consistent Molecular-Orbital Methods. IX. An Extended Gaussian-Type Basis for Molecular-Orbital Studies of Organic Molecules. J. Chem. Phys..

[46] Ünal Y., Nassif W., Özaydin B.C., Sayin K. (2020). Scale factor database for the vibration frequencies calculated in M06-2X, one of the DFT methods. Vib. Spectrosc..

[47] Zhurko G.A. Chemcraft, 1.8. http://www.chemcraftprog.com.

[48] Neugebauer J., Reiher M., Kind C., Hess B.A. (2002). Quantum chemical calculation of vibrational spectra of large molecules?Raman and IR spectra for Buckminsterfullerene. J. Comput. Chem..

[49] Duan G., Zhang C., Li A., Yang X., Lu L., Wang X. (2008). Preparation and characterization of mesoporous zirconia made by using a poly (methyl methacrylate) template. Nanoscale Res. Lett..

[50] Ahmed R.M. (2009). Optical study on poly(methyl methacrylate)/poly(vinyl acetate) blends. Int. J. Photoenergy.

[51] Ghorbel E., Hadriche I., Casalino G., Masmoudi N. (2014). Characterization of thermo-mechanical and fracture behaviors of thermoplastic polymers. Materials.

[52] National Institute of Advanced Industrial Science and Technology, SDBSWeb. https://sdbs.db.aist.go.jp.

[53] Stuart B.H. (2005). Infrared Spectroscopy: Fundamentals and Applications.

[54] Lasch P., Noda I. (2019). Two-Dimensional Correlation Spectroscopy (2D-COS) for Analysis of Spatially Resolved Vibrational Spectra. Appl. Spectrosc..

[55] Noda I., Ozaki Y. (2005). Generalized Two-Dimensional Correlation Spectroscopy in Practice. Two-Dimensional Correlation Spectroscopy. In Applications in Vibrational and Optical Spectroscopy.

[56] Dames C. (2013). Measuring the thermal conductivity of thin films: 3 omega and related electrothermal methods. Annu. Rev. Heat Transf..

[57] MIT: Material Property Database. https://www.mit.edu/~6.777/matprops/pmma.htm.

[58] Sandell S., Maire J., Chávez-Ángel E., Sotomayor Torres C.M., Kristiansen H., Zhang Z., He J. (2020). Enhancement of Thermal Boundary Conductance of Metal–Polymer System. Nanomaterials.

