# Supplementary material for: Application of Synchrotron Radiation-Based Fourier-Transform Infrared Microspectroscopy for Thermal Imaging of Polymer Thin Films
Source: Polymers (Basel). 2023 Jan 19;15(3):536. doi: 10.3390/polym15030536 (PMC9919785; doi:10.3390/polym15030536)
Supplement: Supplementary file 1 [file polymers-15-00536-s001.zip › polymers-2165269-supplementary.pdf]

polymers

Supplementary Materials

Application of Synchrotron Radiation-Based Fourier-Transform Infrared Microspectroscopy for Thermal Imaging of Polymer Thin Films

Emigdio Chavez-Angel [1,*], Ryan C. Ng [1], Susanne Sandell [2], Jianying He [2], Alejandro Castro-Alvarez [3], Clivia M. Sotomayor Torres [1,4] and Martin Kreuzer [5,*]

1 Catalan Institute of Nanoscience and Nanotechnology (ICN2), CSIC and BIST, The Universitat Autònoma de Barcelona Campus, 08193 Barcelona, Spain
2 NTNU Nanomechanical Lab, Department of Structural Engineering, Norwegian University of Science and Technology (NTNU), 7491 Trondheim, Norway
3 Laboratorio de Bioproductos Farmacéuticos y Cosméticos, Centro de Excelencia en Medicina Traslacional, Facultad de Medicina, Universidad de La Frontera, Temuco 4780000, Chile
4 Institución Catalana de Investigación y Estudios Avanzados (ICREA), 08010 Barcelona, Spain
5 CELLS-ALBA, Synchrotron Light Source, 08290 Barcelona, Spain
* Correspondence: emigdio.chavez@icn2.cat (E.C.-A.); mkreuzer@cells.es (M.K.)

Calibration of the Electrical Resistivity of the Metal Wire

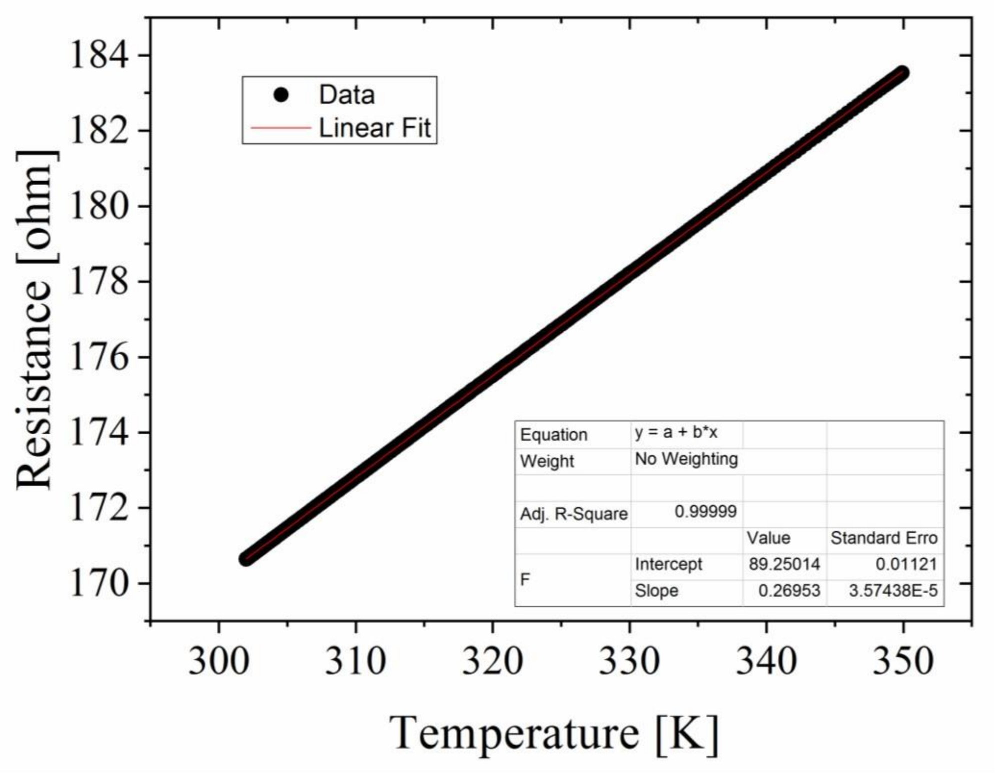

Figure S1. Electrical resistivity of the gold wire measured at different temperatures in a heating stage.

DFT Simulation of the IR Spectra

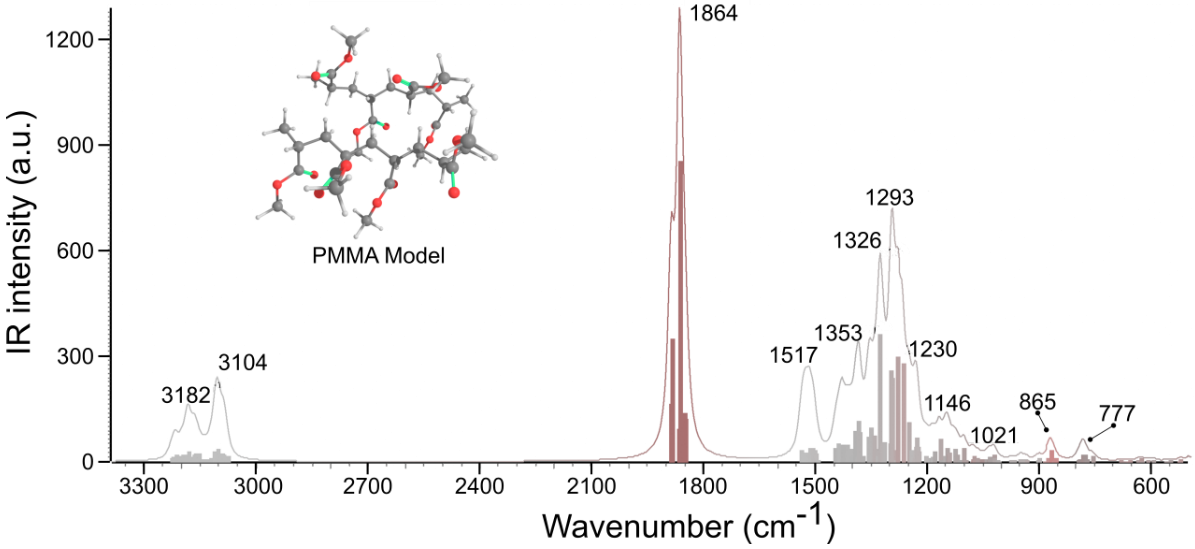

Figure S2. Simulated IR spectra of PMMA molecules.

The PMMA model was created using four methylacrylate units positioned in two rows (inset Figure S2). The model assumes tetrameric PMMA units with atactic tacticity, as this is the most likely tacticity when PMMA is treated at temperatures above 50°C [1]. Then the system was optimized at the M06-2X level [2] with a 6-31G(d) [3] basis set and a scaling factor of 0.947 [4]. The calculations were carried out using Gaussian 16. The frequency calculation was brought to the same level and the absence of negative frequencies in the system was confirmed. The graphs were extracted from the Chemcraft program [5]. The amplitude of the bands (broaden bands) was carried out with the Lorentzian broadening method. The color of the bands is determined by the molecular weight, where the reddish color corresponds to the bands with functional groups associated to oxygen and gray to the bands of carbon atoms.

From the simulation we can observe that the bands are displaced around 100 cm^{-1} compared with the experimental data. Such behavior is normal considering the total size of the cluster used in the simulation compared with a real PMMA chain. It is also to remark that such calculations are independent on temperature [6].

Temperature Dependence of Peak Position, Amplitudes, and Full Width Half Maximum (FWHM)

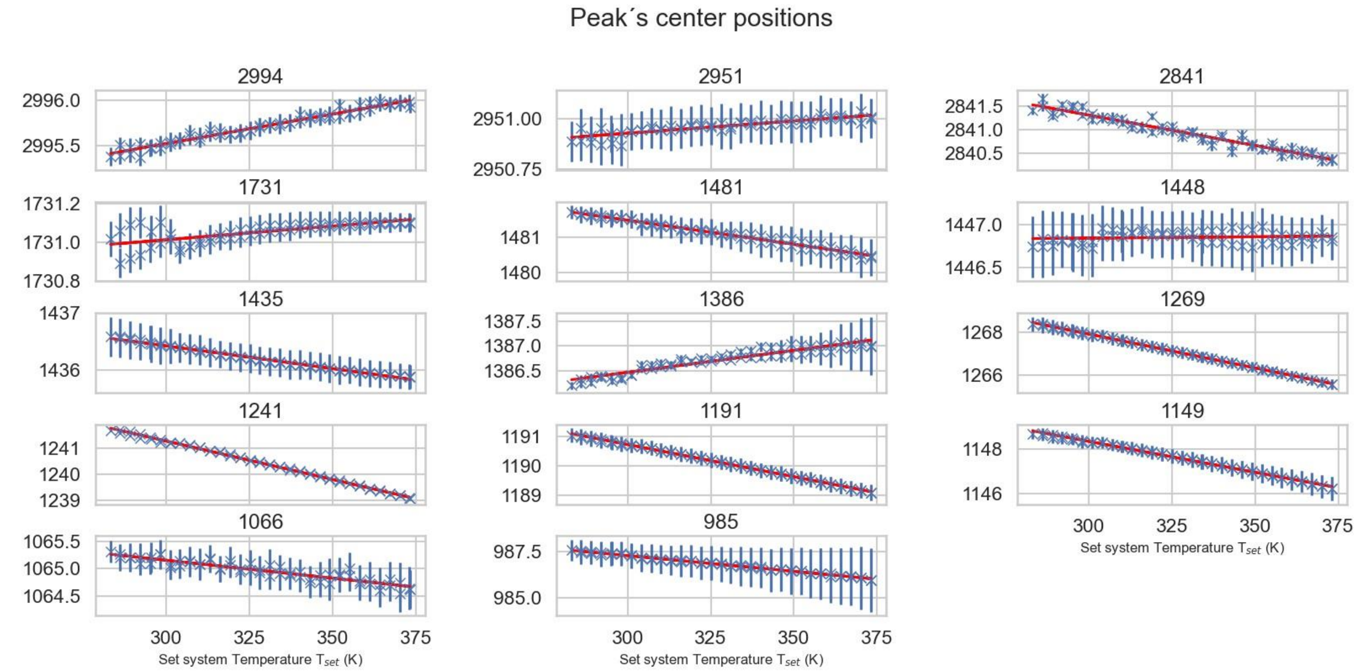

Figure S3. Fitted Gaussian center positions for each PMMA absorbance peak vs temperature.

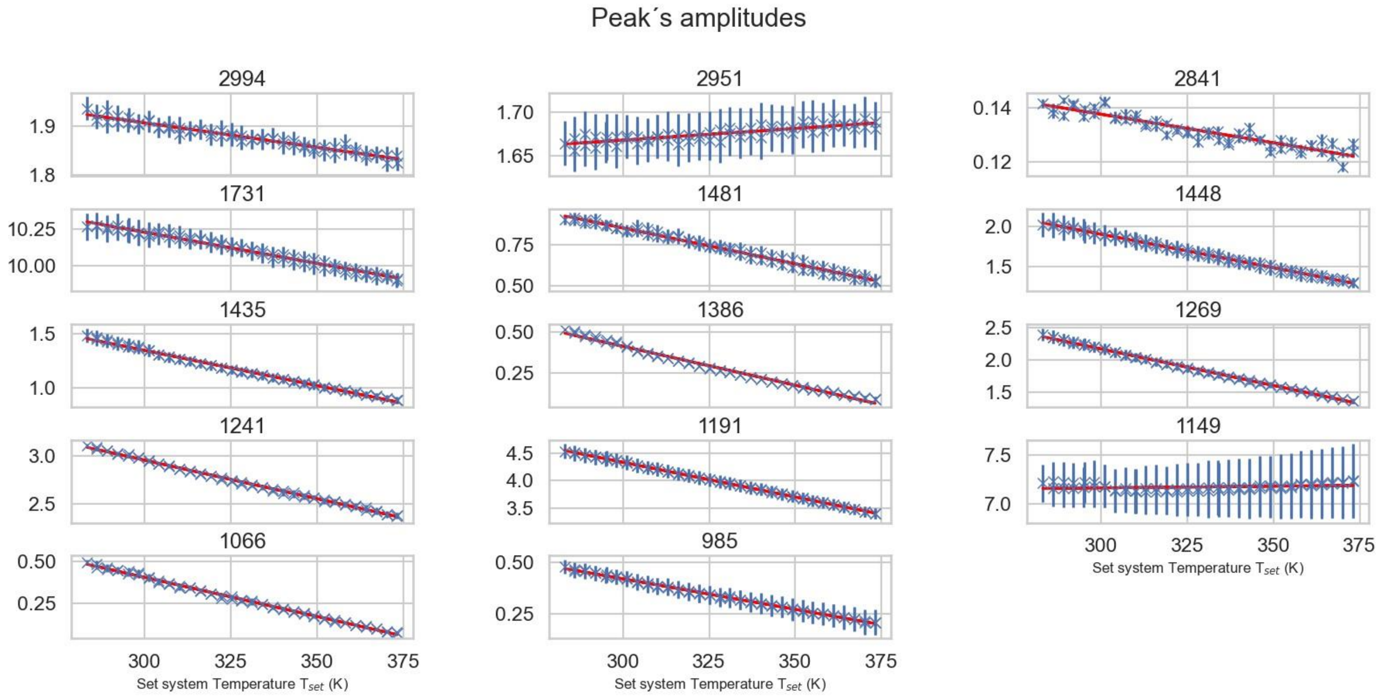

Figure S4. Fitted Gaussian amplitudes for each PMMA absorbance peak vs temperature.

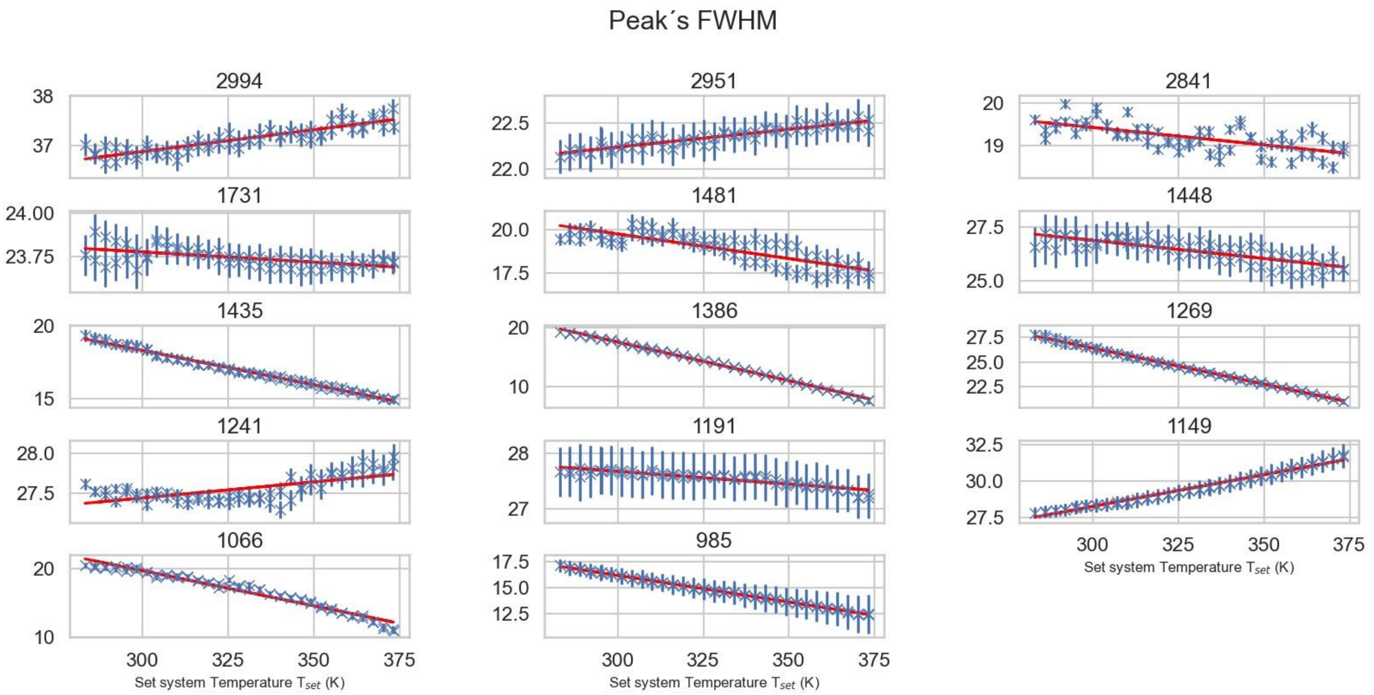

Figure S5. Fitted Gaussian full width at half maximum (FWHM) for each PMMA absorbance peak vs temperature.

Two-Dimensional Correlation Maps

To gain insight into the thermal behavior of the PMMA films, we applied two-dimensional correlation analysis to the FTIR spectra during the heating and cooling process. Two-dimensional correlation spectroscopy (2DCOS) is a mathematical method for analyzing changes in a signal produced by an external perturbation (e.g., a change in temperature, pressure, pH, concentration of mixtures, etc.). To calculate the 2DCOS map we used the temperature as an external perturbation and the spectra dataset was ordered from lowest to highest temperature. The raw spectra were baselined and normalized using the most-intense band (1731 cm^{-1}). The average spectrum was used as a reference spectrum. The 2DCOS analysis was performed with the Mat2dcorr Matlab toolbox [7]. Figure S6 shows the synchronous (a) and asynchronous (b) 2DCOS map for a FTIR frequency window around 1150 cm^{-1}. The respective FTIR-averaged spectra are shown above and in the left part of each frequency window.

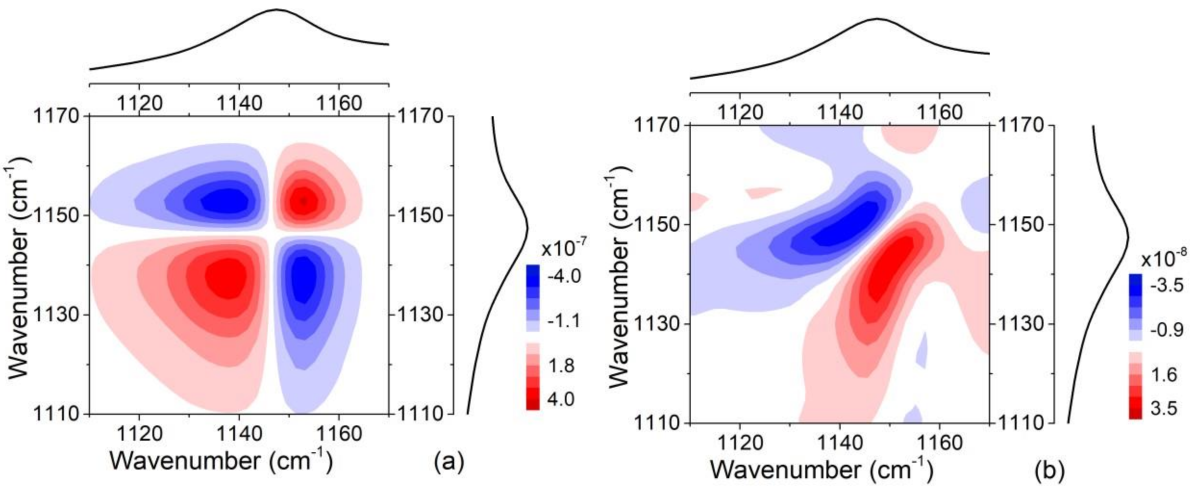

Figure S6. Synchronous (**a**) and asynchronous (**b**) two-dimensional correlation spectra obtained from temperature-dependent FTIR spectra of the PMMA film. The red and blue colors represent positive and negative cross peaks, respectively.

Synchronous spectra show the impact of three coupled effects as the temperature increases: shift in the band position, broadening of the peak, and an increase of the peak intensity of the IR signal. The asymmetrical four-leafed clover pattern indicates the red-shift of the peak is strongly coupled with peak broadening and a decrease in the peak intensity as the temperature increases. The four-leafed clover pattern is composed by two autopeaks and two negative cross peaks centered close to the maximum peak position of the average spectrum. This kind of spectra is very similar to two overlapped bands. However, the butterfly-like pattern of the asynchronous is an indicator of the existence of a shift in the band position [8]. The negative cross peak above the diagonal is an indicator that the band position is red shifting, as was also shown in the fitted values of the peak position. The effect of individual change in band intensity, peak position, band broadening and the coupled effects in the synchronous spectra are shown in Figure S7. The simulation was done by using Lorentzian peaks in an arbitrary spectral region between 1170-1110cm^{-1}and varying the peak intensity (Figure S7a), position (Figure S7b), linewidth broadening (Figure S7c) and the three parameters simultaneously (Figure S7d). The variation of each parameter was taken from the experimental fluctuation of the 1149 cm^{-1} peak.

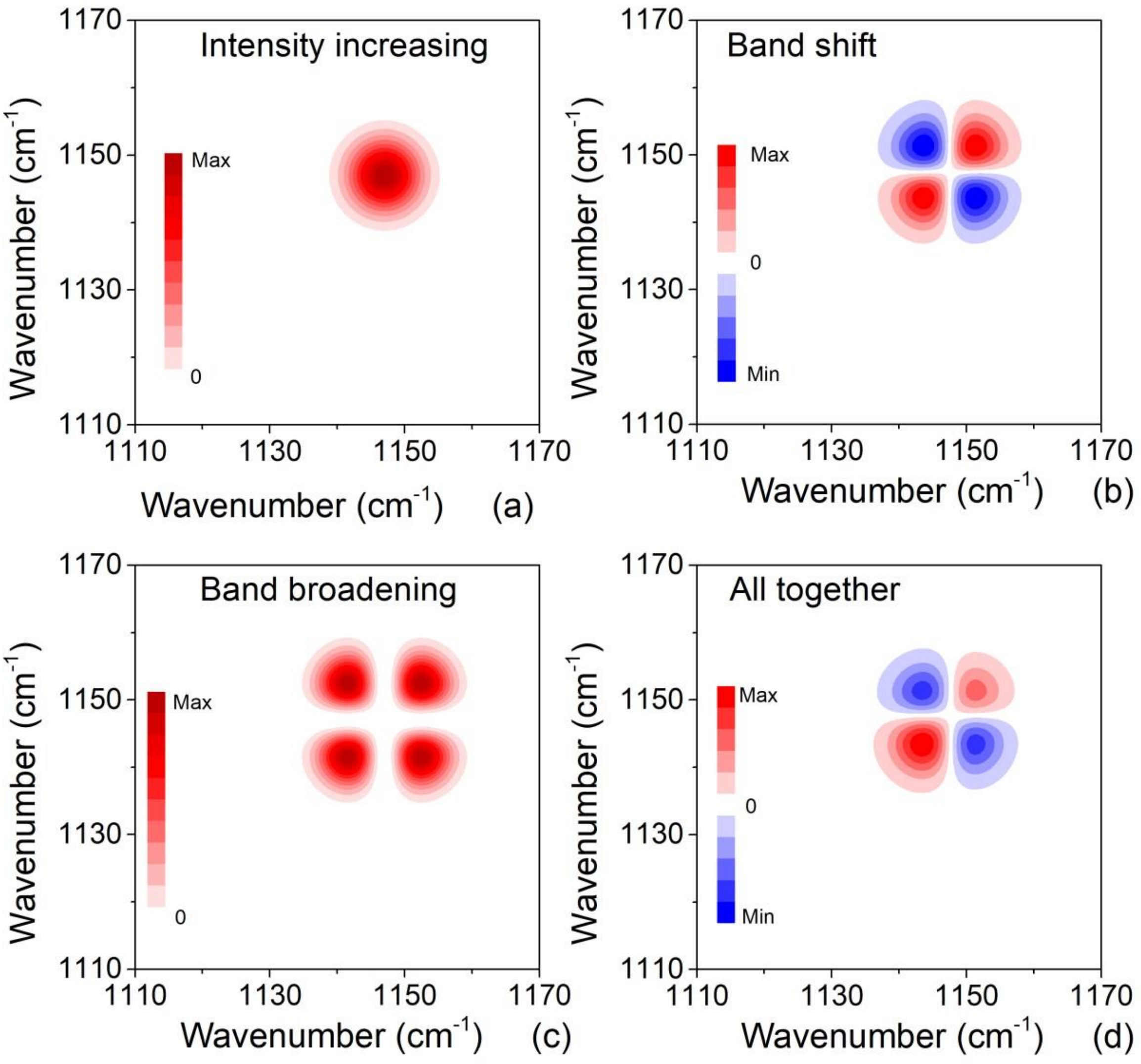

Figure S7. Simulated synchronous two-dimensional correlation spectra centered at ~1149 cm^{-1}. (a) Simulated 2DCOS fixing band position and linewidth and varying the intensity. (b) Simulated 2DCOS fixing band intensity and linewidth and varying the peak position. (c) Simulated 2DCOS fixing band position and intensity and varying the linewidth. (d) Simulated 2DCOS varying band position, linewidth, and intensity.

Fitting Curves

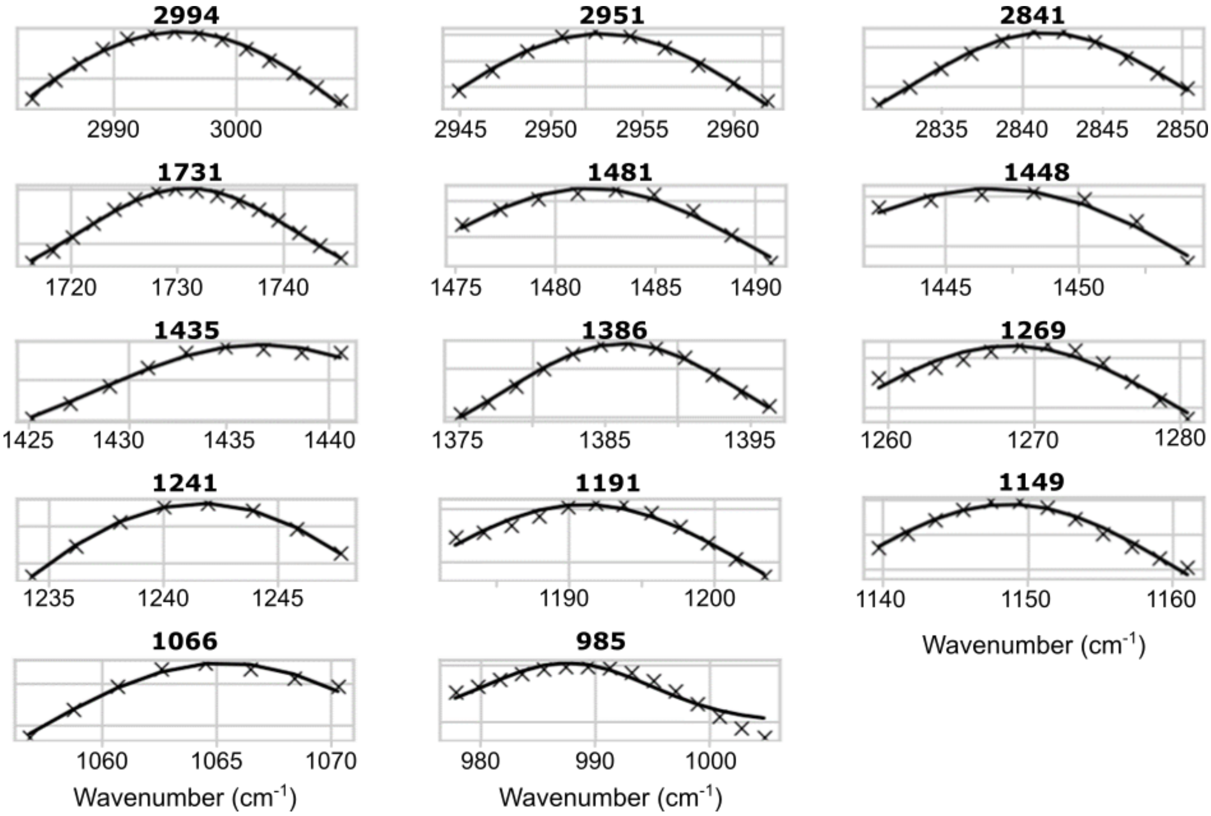

Figure S8. PMMA absorbance peaks at 283 K fitted individually using a Gaussian line shape, showing the data points and the fit as black lines.

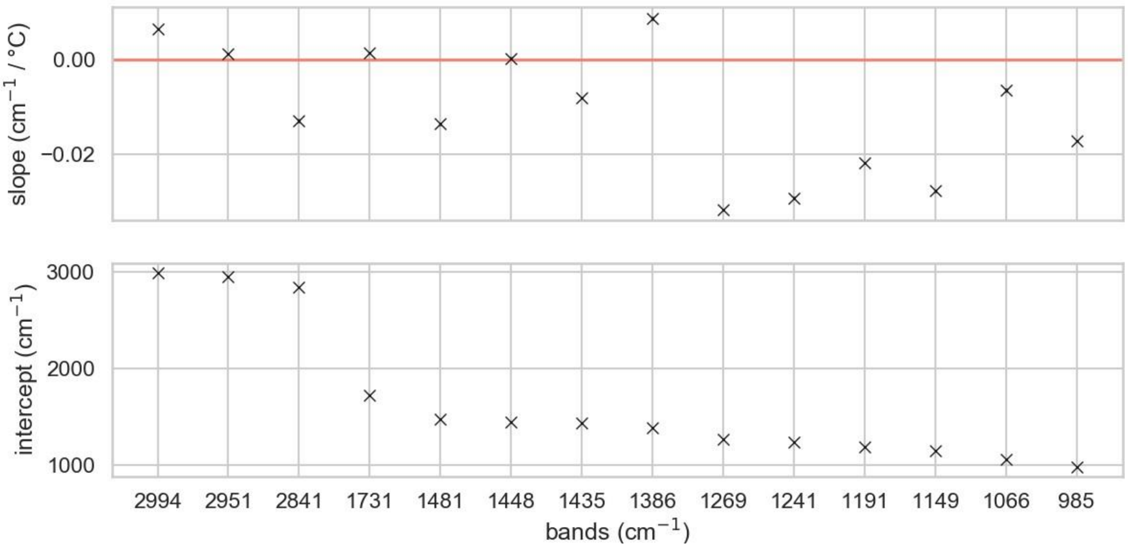

Figure S9. Fitting results for a linear fit to the temperature dependence of the peak center positions for different absorbance peaks. The linear fits are shown in red in Figure S3.

Determination of Thermal Properties

The thermal conductivity was obtaining by solving the heat equation in analogy to the heat spread of a long fin, where the temperature profile in the plane of the film is given by [9]:

$$\Delta T = Q\sqrt{\frac{1}{hkd}}\exp\left[-\sqrt{\frac{h}{kd}}x\right] \quad (1)$$

where Q is power density, h is the "convection coefficient" (which here is thermal boundary conductance (TBC) between polymer and the substrate), d the film thickness, and k the thermal conductivity of the polymer. The thermal conductivity and interphase thermal resistance was estimated from the best fit of Eq. 1 to the experimental data. The fitting was simultaneously done by concatenating the experimental data and the fitting equation through a Kronecker delta function as follows:

$$\Delta T_{Experimental} = (x_{1,i}, T_{1,i}, i\, ; x_{2,j}, T_{2,j}, j\, ; \ldots) \quad (2)$$

$$\Delta T = A\sqrt{\frac{1}{hkd}}\exp\left[-\sqrt{\frac{h}{kd}}x\right]\delta_{1,j} + B\sqrt{\frac{1}{hkd}}\exp\left[-\sqrt{\frac{h}{kd}}x\right]\delta_{2,j} \quad (3)$$

Using this approach, a simultaneous fitting was obtained as shown in Figure S10.

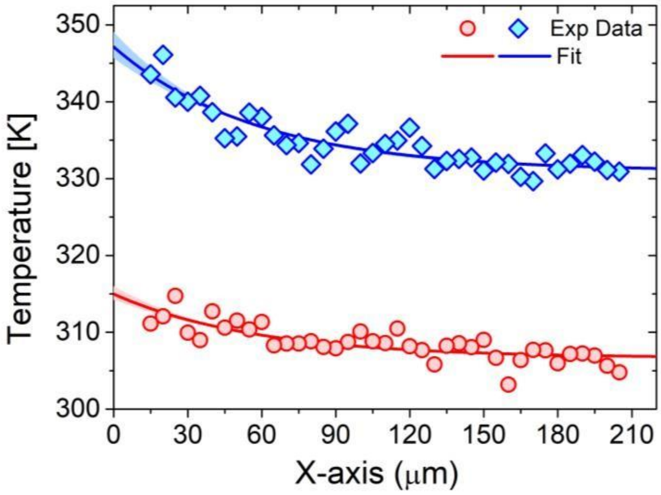

Figure S10. Thermal decay as function of sample position for different temperatures of the heater wire. The solid lines represent the best fit.

The best fit provides k = 0.28 ± 0.11 $WK^{-1}m^{-1}$ and TBC h = 85.30 ± 0.15×10^6 $WK^{-1}m^{-2}$ showing good agreement with the expected values for the thermal conductivity 0.15 < k_{pmma} < 0.25 ($WK^{-1}m^{-1}$) [10] and the same order for the TBC 60 < h < 150 ×10^6 $WK^{-1}m^{-2}$ for the case of PMMA-metal [11].

Principal Component Analysis

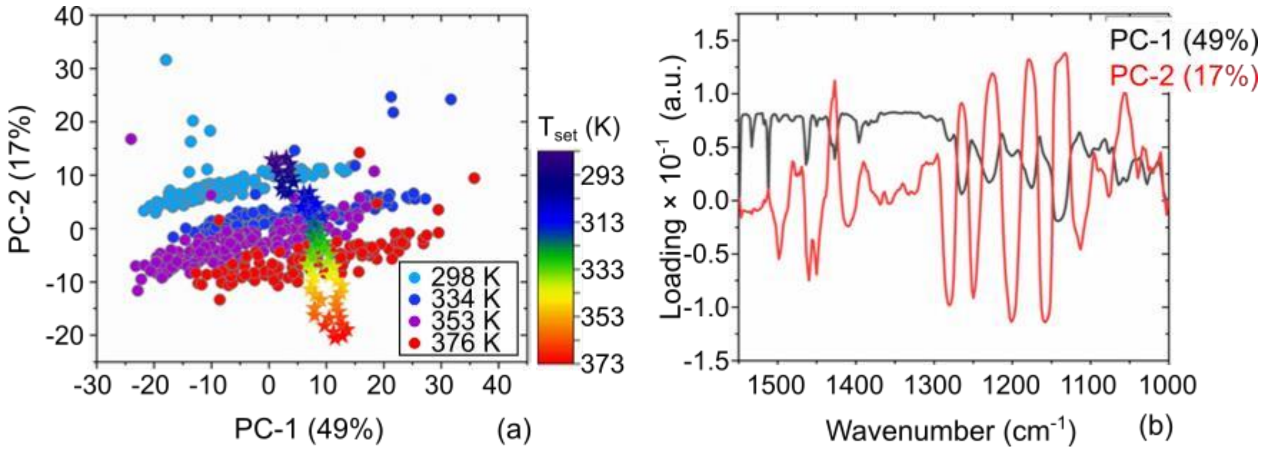

Figure S11. (a) Transferred data (scores plot) of the PCA with the calibration data (stars) and the line scan data points (circle). (b) Loadings plot of the PCA showing PC-1 (black) and PC-2 (red).

Workflow of Machine Learning Algorithms

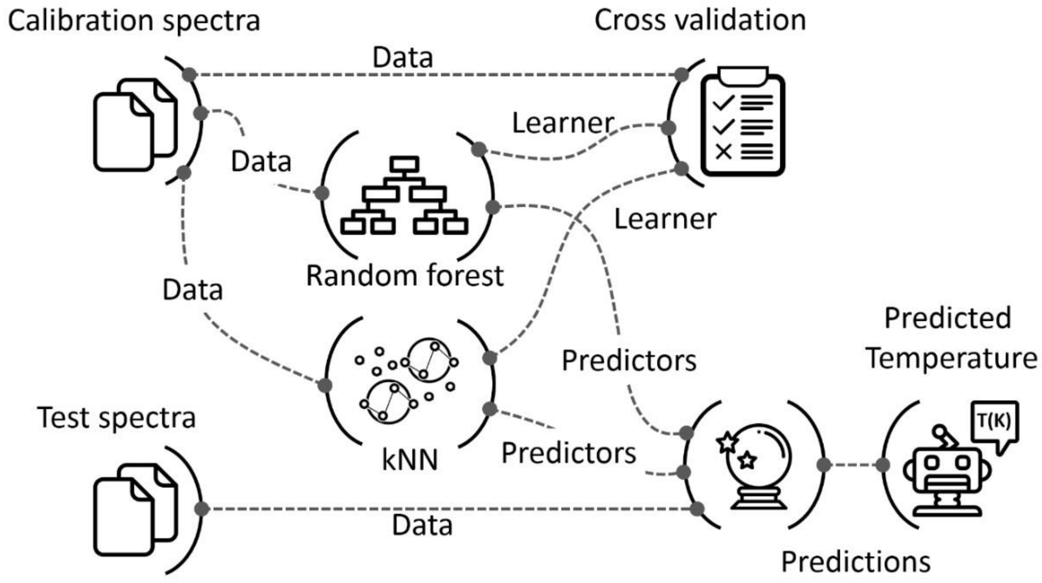

Figure S12. Simplified workflow of the machine learning approach in this work done by Orange [12–14].

References

1. Forte, M.; Silva, R.; Tavares, C.; Silva, R. Is Poly(methyl methacrylate) (PMMA) a Suitable Substrate for ALD?: A Review. *Polymers* **2021**, *13*, 1346, https://doi.org/10.3390/polym13081346.
2. Zhao, Y.; Schultz, N.E.; Truhlar, D.G. Design of Density Functionals by Combining the Method of Constraint Satisfaction with Parametrization for Thermochemistry, Thermochemical Kinetics, and Noncovalent Interactions. *J. Chem. Theory Comput.* **2006**, *2*, 364–382, https://doi.org/10.1021/ct0502763.
3. Ditchfield, R.; Hehre, W.J.; Pople, J.A. Self-Consistent Molecular-Orbital Methods. IX. An Extended Gaussian-Type Basis for Molecular-Orbital Studies of Organic Molecules. *J. Chem. Phys.* **1971**, *54*, 724–728, doi:10.1063/1.1674902.
4. Ünal, Y.; Nassif, W.; Özaydin, B.C.; Sayin, K. Scale factor database for the vibration frequencies calculated in M06-2X, one of the DFT methods. *Vib. Spectrosc.* **2020**, *112*, 103189, https://doi.org/10.1016/j.vibspec.2020.103189.
5. Zhurko, G.A. Chemcraft, 1.8. Available online: http://www.chemcraftprog.com (accessed on 01 December 2022).
6. Neugebauer, J.; Reiher, M.; Kind, C.; Hess, B.A. Quantum chemical calculation of vibrational spectra of large molecules?Raman and IR spectra for Buckminsterfullerene. *J. Comput. Chem.* **2002**, *23*, 895–910, https://doi.org/10.1002/jcc.10089.
7. Lasch, P.; Noda, I. Two-Dimensional Correlation Spectroscopy (2D-COS) for Analysis of Spatially Resolved Vibrational Spectra. *Appl. Spectrosc.* **2019**, *73*, 359–379, https://doi.org/10.1177/0003702818819880.
8. Noda, I.; Ozaki, Y. Generalized Two-Dimensional Correlation Spectroscopy in Practice. In *Two-Dimensional Correlation Spectroscopy. In Applications in Vibrational and Optical Spectroscopy*. John Wiley & Sons, Ltd: Chichester, UK, 2005; pp 47–64.
9. Dames, C. Measuring the thermal conductivity of thin films: 3 omega and related electrothermal methods. *Annu. Rev. Heat Transf.* **2013**, *16*, 7–49. https://doi.org/10.1615/AnnualRevHeatTransfer.v16.20.
10. MIT: Material Property Database. Available online: https://www.mit.edu/~6.777/matprops/pmma.htm (accessed on 01 December 2022).
11. Sandell, S.; Maire, J.; Chávez-Ángel, E.; Sotomayor Torres, C.M.; Kristiansen, H.; Zhang, Z.; He, J. Enhancement of Thermal Boundary Conductance of Metal–Polymer System. *Nanomaterials* **2020**, *10*, 670. https://doi.org/10.3390/nano10040670.
12. Toplak, M.; Read, S.T.; Sandt, C.; Borondics, F.; Vaccari, L.; Byrne, H.J.; Wrobel, T.P. Quasar: Easy Machine Learning for Biospectroscopy. *Cells* **2021**, *10*, 2300. https://doi.org/10.3390/CELLS10092300.
13. Demsar, J.; Curk, T.; Erjavec, A.; Gorup, C.; Hocevar, T.; Milutinovic, M.; Mozina, M.; Polajnar, M.; Toplak, M.; Staric, A.; et al. Orange: Data Mining Toolbox in Python. *J. Mach. Learn. Res.* **2013**, *14*, 2349–2353.
14. Toplak, M.; Birarda, G.; Read, S.; Sandt, C.; Rosendahl, S.M.; Vaccari, L.; Demšar, J.; Borondics, F. Infrared Orange: Connecting Hyperspectral Data with Machine Learning. *Synchrotron Radiat. News* **2017**, *30*, 40–45. https://doi.org/10.1080/08940886.2017.1338424.
